# Longevity and the drift barrier: Bridging the gap between Medawar and Hamilton

**DOI:** 10.1002/evl3.173

**Published:** 2020-05-24

**Authors:** Jussi Lehtonen

**Affiliations:** ^1^ Faculty of Science School of Life and Environmental Sciences The University of Sydney Sydney NSW 2006 Australia

**Keywords:** Evolution of ageing, genetic drift, senescence, theory

## Abstract

Most organisms have finite life spans. The maximum life span of mammals, for example, is at most some years, decades, or centuries. Why not thousands of years or more? Can we explain and predict maximum life spans theoretically, based on other traits of organisms and associated ecological constraints? Existing theory provides reasons for the prevalence of ageing, but making explicit quantitative predictions of life spans is difficult. Here, I show that there are important unappreciated differences between two backbones of the theory of senescence: Peter Medawar's verbal model, and William Hamilton's subsequent mathematical model. I construct a mathematical model corresponding more closely to Medawar's verbal description, incorporating mutations of large effect and finite population size. In this model, the drift barrier provides a standard by which the limits of natural selection on age‐specific mutations can be measured. The resulting model reveals an approximate quantitative explanation for typical maximum life spans. Although maximum life span is expected to increase with population size, it does so extremely slowly, so that even the largest populations imaginable have limited ability to maintain long life spans. Extreme life spans that are observed in some organisms are explicable when indefinite growth or clonal reproduction is included in the model.

**Impact Summary**

Our mortality is a topic that captures the attention of scientists and the public alike. What are the ultimate causes of ageing and eventual death? Can we predict the longevity of organisms based on their traits? We typically take it for granted that living things age and do not live forever, and evolutionary theory provides theoretical understanding of why organisms age. We also often take it for granted that maximum life spans of familiar organisms are not only limited, but they fall in some specific range. The maximum life span of mammals, for example, is at most some years, decades, or centuries. Why not thousands or tens of thousands of years, or more? This aspect of the evolution of longevity is more elusive. Even if theories can tell us reasons for why living things must age, they do not offer an easy path to see where limits to life span might lie and why. In other words, existing theory provides reasons for the prevalence of ageing, but making explicit quantitative predictions of life spans is difficult. I develop a mathematical theory that accounts for two factors that are missing from much of earlier work: mutations of large effect, and finite population size. This combination in turn makes it possible to link a concept called the “drift barrier” from evolutionary theory to the evolution of ageing. The drift barrier refers to a limit where the power of random genetic drift overcomes the power of natural selection. Using such a model setup, I calculate a drift barrier for a limiting age where natural selection can no longer remove even deadly mutations from the population. This drift barrier is an approximate theoretical limit for maximum life spans that natural selection can support. The theory yields results in the observed range for mammals, and suggests directions for empirical tests.

The prevalence of senescence and intrinsically limited life span is one of the oldest conundrums in biology. Weismann proposed an evolutionary explanation for ageing and limited longevity as far back as 1891 (Weismann 1891), but arguably the most important contributions to the topic appeared in the 1950s–1960s in work by Medawar (1952), Williams (1957), and Hamilton (1966)—articles that are still among the most influential in evolutionary biology several decades later. The theory laid out by Medawar is often called the “mutation accumulation” theory for the evolution of senescence, and Hamilton's contribution provides the mathematical tools that can be used to formalize this verbal theory and give it a more rigorous foundation. In other words, Hamilton's paper is said to provide a formal and quantitatively explicit analysis of Medawar's forces of natural selection (e.g., Rose et al. 2007, Sherratt & Wilkinson 2009).

Here, I argue that although this description of the link between Medawar and Hamilton is justified and true, it is only a partial truth. First, although Hamilton's model was a major breakthrough, there are two significant aspects of Medawar's verbal model that Hamilton's mathematical approach cannot address. Second, although Hamilton's model is quantitative in the sense that it provides a mathematical method to analyze age‐specific forces of selection, it does not provide a clear way to predict the absolute length of life spans that we should expect to evolve. For example, it yields a convincing reason for why mammals should age, but it remains unclear why mammals should live a few years, decades, or two centuries at most, rather than thousands or tens of thousands of years. Constructing a mathematical model that more closely follows the assumptions of the verbal example that Medawar (1952) presented takes us toward a resolution to the second issue (prediction of absolute life spans).

I will begin by recapitulating a central example that Medawar (1952) presented to drive home his argument. I will then explain why Hamilton's (1966) model cannot address this example, and derive a complementary model that can. Medawar (1952) proposed that a decline in survival with age can escape natural selection because older age classes are increasingly invisible to selection. Central to the argument was a thought experiment with a hypothetical stock of 1000 test tubes that are randomly broken at a rate of 10% per month. Furthermore, the tubes somehow reproduce themselves at a similar rate to maintain their numbers constant. Medawar argued that even if the vulnerability of the test tubes to breakage does not increase with age (i.e., the test tubes do not senesce), they are nevertheless more likely to have been randomly broken by the time they would reach old age simply because they have spent more time under constant risk of breakage. Now, instead of a population of unchanging, nonsenescent test tubes, imagine that the test tubes carry a stopwatch that is started at birth and a self‐destruct device that shatters the tubes when the stopwatch reaches a given, large enough reading (say, 100 months). Despite its seemingly drastic effect, few (if any) tubes will in fact be affected by this self‐destruct device, because they have very likely been shattered by random breakage before their time is up. The point Medawar was making with this example is that if, instead of test tubes, we consider a population of living organisms, a lethal mutation causing such age‐specific self‐destruction would have very little effect because it would hardly ever be expressed before the organism is dead anyway due to extrinsic mortality, which must always be present in a stationary or near‐stationary population. Therefore, even a lethal mutation with sufficiently late age of onset will be very weakly selected against if it appears in an initially nonsenescent, immortal population.

Where does this deviate from Hamilton's (1966) mathematical model? There are two salient points. First, the nature of the deleterious mutations is a major departure. Hamilton's model is based on the mathematical method of implicit differentiation and thus limited to mutations of very small effect, in sharp contrast to the lethal mutation in Medawar's verbal example. Second, Hamilton's model and those elaborating on it do not typically account for effects of finite populations, in contrast with the stationary population of 1000 individuals in Medawar's example (a noteable exception here is the model and empirical analysis of Lohr et al. 2014, where finite population size is accounted for, but mutation effects remain small as in Hamilton's 1966 model). To be clear, the intention is not to claim that one of these classic papers is better than the other: Medawar's analysis has known limitations (inherited from the brief remarks of Fisher 1930; see, e.g., Charlesworth 2000), and it would not be fruitful to rank a verbal and mathematical model in this way in any case. Nor is the intention to claim that researchers are not aware of mutations of both small and large effect, or of finite populations—the point is simply that the two models differ on these points. The assumptions of Hamilton (1966) are not “wrong”—they are the kinds of simplifying assumptions that are often needed for a tractable analysis. But they do prevent us from seeing fundamental results that arise when a mathematical model is constructed to explicitly follow Medawar's verbal account with lethal mutations and a population that is finite and fixed in size. Most importantly, the new model makes explicit predictions about maximum life span as a function of simple life history and population parameters. For example, it suggests a simple explanation for typical mammalian life spans, something that previous theory has limited ability to do.

## Methods

### ANALYTICAL MODEL

The starting point is a biological parallel of Medawar's test tube population: a hypothetical population of initially non‐senescent organisms, such that a newly matured individual is indistinguishable from one that has successfully reproduced several times. We then ask: can a lethal mutation (i.e., one that shatters a test tube) that is expressed at a specific age spread in a finite population of effective size $N_{e}$ (see Table 1 for notation), and if so, at what age? The age at which this can happen is determined by the drift barrier (Kimura 1962; Li 1978; Kimura 1984; Lynch 2007), where the power of random genetic drift overcomes the power of selection. Analogous to the drift barrier imposing a limit on cellular perfection (Lynch 2012) and a lower bound on mutation rates (Lynch et al. 2016), here the drift barrier imposes an upper bound on life span. In the drift barrier hypothesis for mutation rate evolution, natural selection primarily selects for reduced mutation rates, whereas random genetic drift (which depends on population size) prevents the maintenance of zero mutation rates (Lynch et al. 2016). In the present model, natural selection primarily selects for the maintenance of longer life spans, but random genetic drift (again, dependent on population size) prevents the maintenance of infinite life spans. This approach allows us to relate the limits of maximum life span quantitatively to population size and life history characteristics.

**Table 1:** Notation used in the model

Notation	Name of parameter, variable, or function	Notes
$N_{e}$	Effective population size	
*b*	Age at first reproduction	Age at which individuals are capable of reproduction for the first time
σ	Juvenile survival	Probability of a newborn offspring surviving to reproductive age
μ	Adult mortality for continuous reproduction	Instantaneous mortality rate of reproductive individuals
*f*	Fecundity	Rate of reproduction in female offspring per mother per year. Denoted *f*(*t*) if age specific.
*l*(*t*)	Survival probability	Probability of survival from birth to age *t*, taking into account both juvenile and adult survival
*σf*	Recruitment	Rate of offspring surviving to maturity per mother per year (i.e., juvenile survival multiplied by fecundity)

The method is first introduced with a life history model that corresponds to Medawar's example (Medawar 1952) and serves as an approximation to the life histories of many mammals and birds (Charlesworth 2001; Oli & Dobson 2003; however, note that it is not intended to be a model of human life history where female reproduction typically ceases relatively early in life). The life cycle of the organism is divided into two phases: a maturation phase of length *b*, which a newborn survives with probability σ, after which the newly matured individual enters a pool of adults competing for reproductive opportunities. She thereafter reproduces with fecundity *f* per time unit, and the reproductive phase continues until she is eventually unsuccessful and dies.

Now, again following Medawar, consider a newly introduced mutation that is lethal at age *x* and the selection coefficient against such a mutation in an initially nonsenescent population. Obviously not all mutations are lethal in nature. However, aside from being faithful to Medawar's example, the role of this “test mutation” in the current model is to provide a limiting case for computing an age beyond which the population is outside the reach of natural selection. If we can find a limit where lethal, age‐specific mutations become invisible to selection, we can be sure that beyond that limit any age‐specific mutation with less drastic effects is also invisible to selection. In this sense, the lethal mutation is a computational aid rather than an exact biological assumption.

In the ancestral nonsenescent population, adult extrinsic mortality (μ) is independent of age, so that survival to age *t* > *b* is $l\left(t\right)=\sigma e^{-\mu (t-b)}$ (the product of juvenile survival and negative exponential survival probability arising from constant mortality in the reproductive phase). Age‐specific fecundity is f(t)=f for *t* > *b* and 0 otherwise. A stationary population (as in Medawar's example) then implies $\int_{0}^{\infty }l\left(t\right)f\left(t\right)dt=\int_{b}^{\infty }\sigma e^{-\mu (t-b)}fdt=\frac{\sigma f}{\mu }=1$, from which it can be solved that μ=σf (i.e., recruitment into the adult population equals adult mortality). This constraint does not imply commitment to a specific causal relationship among μ, σ, and *f*: it results from the reasonable assumption that over long, evolutionary timescales the population is close to stationary (Fisher 1930; Hamilton 1966; Charlesworth & Williamson 1975), implying that adult mortality must be in balance with recruitment regardless of causal relationship. There may of course be complicated underlying causal processes that keep population size in check, but the assumption of a stationary population links adult mortality inexorably to recruitment regardless of the nature and complexity of such processes (see also, e.g., Cohen et al. 2020). Under the present assumptions, an observation of the value for either recruitment or adult mortality implies the value of the other, and for the purposes of the model it is not necessary to know the processes keeping them in balance. Now consider the fate of the mutant who dies at age *x* if she has survived that far. The expected difference in her lifetime reproductive success relative to the wild‐type is

(1) $$\begin{array}{c} s=\left\{\begin{array}{cc} -\int_{b}^{\infty }\sigma e^{-\sigma f\left(t-b\right)}fdt=-1 & x\leq b\\ -\int_{x}^{\infty }\sigma e^{-\sigma f\left(t-b\right)}fdt=-e^{-\sigma f(x-b)}=-e^{-\mu \left(x-b\right)} & x>b \end{array}\right.. \end{array}$$

Equation 1 is the selection coefficient against such a mutation. A mutation that is deadly before maturation can never be fixed in a population (s=−1), but the selection coefficient decreases thereafter, and the decrease is rapid if adult extrinsic mortality is high. In a hypothetical infinite population, such a mutation could not go to fixation no matter how small the negative selection coefficient, but a finite population behaves in a fundamentally different way. Classic results on mutation fixation in finite populations (Kimura 1962) have a remarkably simple corollary (Li 1978; Kimura 1984): a deleterious mutation can spread over the population when $|s|<1/N_{e}$ (the drift barrier: Lynch 2007). Therefore, the approximate limiting age *x* is found by solving $e^{-\sigma f(x-b)}=e^{-\mu (x-b)}=1/N_{e}$, which obtains

(2) $$x=b+\frac{\ln\left(N_{e}\right)}{\mu }=b+\frac{\ln\left(N_{e}\right)}{\sigma f}.$$

Equation 2 describes a temporal drift barrier for age‐specific mutations, written in alternative and complementary ways. The importance of the form $b+\frac{\ln(N_{e})}{\sigma f}$ is that both *b* and *f* are traits of the organism, whereas μ is under the organism's control to a lesser extent. The equation $x=b+\frac{\ln(N_{e})}{\sigma f}$ is therefore more concretely tied to the phenotype of the organism than the alternative form. The discrete counterpart of this equation is

(3) $$x=b+\frac{\ln\left(N_{e}\right)}{\ln\left(1+\sigma f\right)}.$$

See Supporting Information for derivation of equation 3. Equation 2 is more transparent about the predicted correlations, whereas equation 3 is more appropriate for organisms with seasonal reproduction, and more efficient to simulate (see next section). Apart from the difference in reproductive timing, their underlying logic is exactly the same, they predict similar correlations, and coincide quantitatively when σf is small.

### SIMULATION MODELS

The aim of the simulations is to confirm the logic of the analytical models and to relax some assumptions made in their derivation. Factors that are of particular interest here are (i) the applicability of the drift barrier concept to an age‐structured population and the robustness of the model when this age‐structure subtly changes as a lethal mutation invades, (ii) robustness of the model when multiple mutations of varying effect sizes exist in the population simultaneously, (iii) a preliminary check for robustness in the face of population size fluctuations, and (iv) the effect of parallel reproductive pathways where one “rejuvenates” the offspring and one may do so to a lesser extent (e.g., gametic vs. budding reproduction in organisms such as hydra—see Discussion). The simulation results arise organically out of a random process without reference to theory on fixation or the drift barrier. A brief description is given here; full simulation code with comments is provided in the Supporting Information.

**Figure 1:**
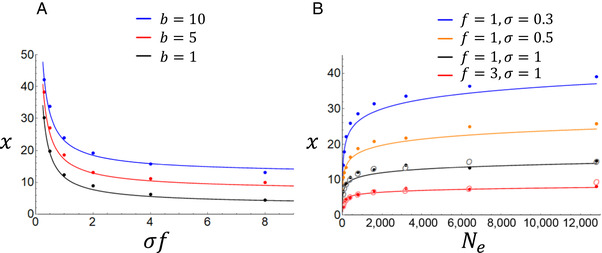
The effects of recruitment, age at first reproduction, and population size on the evolution of maximum life span. The curves are analytical results based on the discrete equation 3, which is more efficient to simulate than the continuous equation 2. Dots are results of simulations that intentionally violate assumptions of the analytical model to check for robustness (see model description in the main text and code in the SI). Mutations arise in every generation so that there are always multiple alleles in the population, and each mutation can increase or decrease life span by a randomly picked amount proportional to current life span. The gamma distribution is parameterised so that on average 1 mutation in 100 is advantageous (i.e., increases maximum life span). However, all mutations still result in certain death at the age thus determined; this assumption is relaxed in Figure 2. Each instance of the simulation was run for 1 million generations (panel A) or 4 million generations (panel B, to account for larger population sizes) in multiples of generation time, and the coloured dots indicate averages of the last 30% of these generations. Note that the curves in panel A are not exact vertically shifted copies of each other as one might first expect from equations (2), (3). The reason for this is that age at first reproduction alters $N_{e}$ when overlapping generations are present. $N_{e}$ for the analytical results was calculated following Hill (1979). In panel A, the adult population size is 1600 individuals, and in panel *B* age at first reproduction is 1. The open circles in panel B are simulation results where fluctuations of large magnitude in population size were allowed around the mean (see Supporting Information). Population persistence in the presence of these fluctuations requires sufficiently high per‐capita recruitment, hence these results are restricted to the two lowest curves in panel B.

Central to all simulations here is a population where the number of adult individuals is regulated so that it stays fixed in size (most simulation figures) or fluctuates around a specified mean (open circles in Fig. 1). The adult population reproduces simultaneously at discrete intervals, corresponding to the analytical equation (3). Adult population size, fecundity (f), juvenile survival (σ), age at first reproduction (*b*), and the shape of the mutation distribution are central features that can be altered. Adult mortality arises implicitly at population regulation and is not explicitly specified. A simulation could be set up with alternative parametrizations and alternative ways of regulating population size, but the aim here is to relate results explicitly to equation 3 and its variables. In each generation, all adults reproduce and some juveniles survive to enter the adult phase, but prior to the next reproductive event the population is culled back to its fixed size from the juveniles that have matured in the current generation and adults surviving from previous generations. The age of every individual is tracked, and if they carry age‐specific deleterious mutations their survival probability at population culling is altered by these mutations (in the case of lethal mutations it become strictly zero). In Figure 3B, offspring reproduced via the budding pathway “inherit” their parent's age, whereas in all other cases offspring are born at age 0 (see Discussion for biological explanation). Note that adult population size is not necessarily equal to $N_{e}$ in an age‐structured population (Hill 1979) and must be computed according to each model parametrization (see Fig. 1A and legend for an example of this effect).

In all simulations, a new mutation arises in one randomly selected parent in every generation. The aim is not to mimic a real mutation rate but to guarantee that multiple mutations are typically present in the population simultaneously, thus ensuring that results are not dependent on a sequence of temporally distinct mutation fixations or on a low mutation rate. Mutations in all simulations are picked from the gamma distribution (the most common mathematical distribution used to model the distribution of mutation fitness effects: Eyre‐Walker & Keightley 2007).

In Figures 1 and 3B, all mutations are lethal, but their age of onset (i.e., maximum life span) varies. When a mutation arises in a single individual, its value is picked from the gamma distribution, truncated to maximum value 2. Maximum longevity is multiplied by this value, so the mutation is deleterious in the range 0–1 and beneficial when >1. The gamma distribution for Figures 1 and 3B is parameterized with shape and scale parameters 1 and 0.218, respectively, resulting in a distribution where approximately 1 in every 100 mutations is beneficial (see Fig. S1 for a visualisation of mutation distributions). For the results indicated with open circles in Figure 1B, adult population size was forced to rapidly fluctuate (fluctuations visualised in Fig. S2, using code shown in the Supporting Information).

**Figure 2:**
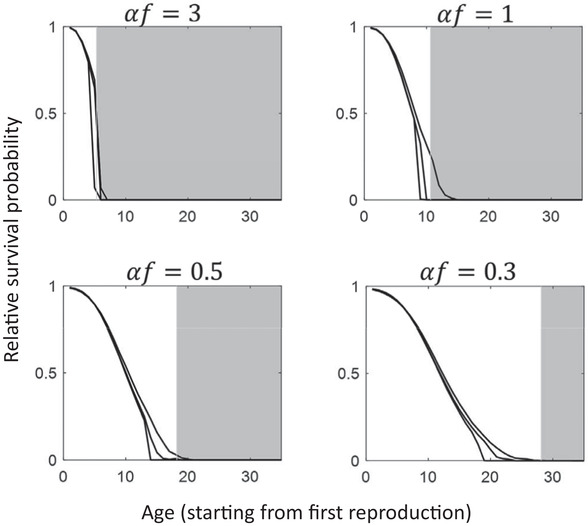
Simulation results and the selection shadow as predicted by equation 3. In contrast to Figure 1, mutations are now typically not lethal, and instead alter age‐specific survival probability at population regulation. A mutation is picked from a gamma distribution (Eyre‐Walker & Keightley 2007) in every generation as in Figure 1, but manifests its effect in a very different way (see methods and Supporting Information). Simulations were run for 1 million generations, and the figures represent the evolved age‐specific survival weightings averaged over the last 30% of these generations. A weighting of 1 is the maximum value (i.e., highest possible survival probability), whereas a weighting of 0 implies certain death at population regulation. The gray zone illustrates the ages where the analytical equation 3 predicts drift to overwhelm selection (hence expected to be an approximate limit to longevity). The three partially overlapping curves within each panel result from mutation distributions parameterized so that on average 1 in 200, 1 in 100, and 1 in 10 mutations are beneficial, moving from leftmost to rightmost curve within each panel (see methods and Supporting Information for details and illustrations of mutation distributions). In all panels, effective population size is 1600 individuals and age at first reproduction is 1 (however, only adult life span is included in the *x*‐axis).

In the simulations underlying Figure 2, the main difference in comparison to Figure 1 is that although a mutation's effect is still picked from the gamma distribution, mutations are now typically not lethal, thus relaxing another simplifying assumption of the analytical model. Instead of altering life span directly, mutations now alter age‐specific relative survival probability at population regulation, whereas the age of onset of the mutation as well as the time‐window (i.e., how long the effect of the mutation persists after age of onset) is separately picked from a uniform distribution (range 1–50 for onset and 0–25 for window). This setup allows the evolution of gradual senescence, in contrast to Figure 1 where maximum life span is directly altered by mutations. The gamma distribution for mutations in Figure 2 is shifted and truncated so values range from –1 to 1 (thus either adding or subtracting from age‐specific survival probability), using shape parameter 1 and scale parameters 0.458, 0.218, and 0.189. These parameters correspond to 1 mutation in 10, 100, and 200, respectively being beneficial (see Fig. S1).

Overall, the objective of these simulations is not to aim for maximal realism, but instead to check whether the relatively simple analytical model is robust to some major deviations from its simplifying assumptions. See figure legends and the full simulation code in the Supporting Information for further details on the simulations.

## Results and Discussion

Any deleterious mutation with effects confined beyond age *x* determined by equations (2), (3) is effectively invisible to selection, as is a beneficial mutation that “repairs” the effects of such a deleterious mutation. Contrarily, below the limit *x*, sufficiently deleterious mutations can be purged and sufficiently beneficial mutations can be fixed by selection. As an upper limit, the temporal drift barrier described by these equations is therefore not strictly limited to the idealised lethal test mutation, but approximately true for a much wider range of mutation distributions. If longevity is temporarily pushed slightly below *x* by deleterious mutations, rare advantageous mutations become visible to selection and increase longevity. If a rare advantageous mutation pushes longevity above the limit, deleterious mutations can easily take over provided they are more common than advantageous ones. There is therefore no strict equilibrium, but longevity is expected to fluctuate near *x*.

The long‐term average resulting from this process will depend to some extent on the distribution of mutation effects. As is the case with many evolutionary models, it is trivial to define unrealistic, extreme mutation distributions where equations (2), (3) will fail to have predictive value. At one extreme, a distribution restricted to advantageous mutations will maintain (or inevitably drift toward) unlimited life spans. At the other, a distribution restricted to deleterious mutations where each subsequent mutation decreases fitness by an increment ≪1/*N*_*e*_ can in principle erode any trait—age‐specific or not—to oblivion. These limitations are of course not specific to the current model, but examples of the more general principle that evolutionary models must commonly specify a reasonable range of possible mutations for their results to hold. Specific analytical models could be constructed for some mutation distributions, and it may be useful to do so for some cases (see, e.g., Lynch 2012 for an example of a drift barrier model with a specific mutation distribution). But for the present argument, this would lead to multiple distribution‐specific submodels, none of which would be likely to be exactly true in nature. The approximate limits given by equations (2), (3) are therefore arguably reasonable initial approximations in addition to being simple and transparent. It is nevertheless of interest to determine whether these equations hold, at least approximately, for some biologically reasonable mutation distributions. Figures 1, 2 apply the gamma distribution (Eyre‐Walker & Keightley 2007) to a range of examples, and they also show that the result holds to a good approximation if mutation rate is high so that several mutations exist in the population simultaneously. Furthermore, Figure 1B indicates that major fluctuations in population size around the mean do not in themselves compromise the results either. In Figure 2, mutations affect the age‐specific relative probability of survival during population regulation, rather than directly altering maximum life span as in Figure 1. It should be noted that the equations derived in this article apply as an approximate upper limit even if mutations have beneficial pleiotropic effects at earlier ages (Medawar 1952; Williams 1957; Kirkwood & Rose 1991) because such effects make the mutation less deleterious overall, shifting the limit toward younger ages. In other words, if a mutation that is lethal at 100 years cannot be purged by selection, then we can be certain that a mutation that is lethal at 100 years and additionally has some positive effect at age 10 cannot be purged by selection either.

The drift barrier described by equations (2), (3) is very different from the “walls of death” in previous studies, a concept based on assumptions of all mutations affecting survival being deleterious (Tuljapurkar 1997; Wachter et al. 2013) and on infinite population size (Wachter et al. 2013). Contrarily, the central requirements for equations (2), (3) to hold approximately are that mutations with age‐specific effects exist (as is assumed in much of previous theoretical work, but see, e.g., Cohen 2004, Kirkwood & Melov 2011, and Wensink 2013 for critical discussion of this assumption), that they are more commonly deleterious than advantageous, and that population size is finite.

Figures 1, 2 are not intended to cover all the bases but rather to examine the most obvious deviations from model assumptions such as variation in the mutation distribution, multiple simultaneously segregating mutations, and fluctuating population size. The figures indicate that equations (2), (3), although approximate, are relatively robust. Future theoretical work could examine in more detail the extent to which results may be altered by factors such as variation in the mutation distribution, life history, regulation and fluctuation of the population, and recombination.

### IMPLICATIONS OF EQUATIONS (2), (3)

Equations (2), (3) illuminate several aspects of the evolution of life span. The negative exponential nature of cumulative survival probability (eq. 1) and the resulting logarithm of population size (eqs. (2), (3)) demonstrate the inefficiency of natural selection at keeping out even the most deleterious age‐specific mutations. In this context, an infinite population is a poor mathematical approximation of any population that could hypothetically ever exist on the planet. There are approximately $10^{50}$ atoms in the earth (Wolfram Research 2020), a number vastly higher than the size of any biological population. Although the logarithmic function has no upper bound, it increases extremely slowly: $\ln(10^{50})$ is only approximately 115. Therefore, although population size is expected to explain some variation in longevity (and has been shown to do so; Lohr et al. 2014), it can also be thought of as a limiting factor whereby realistic effective population sizes place an upper bound on longevity. Humans, for example, have a relatively low $N_{e}$ of approximately 10,400 (Charlesworth 2009), whereas $N_{e}$ for the house mouse is estimated to be 580,000 (Halligan et al. 2010). Yet the difference in $\ln(N_{e})$ is quite small: 9.2 (human) versus 13.3 (house mouse). Assuming that $N_{e}$ for most mammals is in the range 1000–1000,000, $\ln(N_{e})$ will consequently vary between 7 and 14, only a twofold difference between the minimum and maximum. $N_{e}$ can therefore greatly limit life span even in very large populations. Moreover, the slow increase of $\ln(N_{e})$ to some extent compensates for the approximate nature of the drift barrier. As noted by Li (1978), “*the dichotomy between* $s\leq \frac{1}{N}$ *and* $s>\frac{1}{N}$ *is somewhat arbitrary*,” and in reality there is no exact and absolute limit for the selection coefficient at which a barrier emerges. But because $N_{e}$ appears inside the logarithm in equations (2), (3), the inherent fuzziness of the drift barrier is “compressed,” so that it provides a more solid and accurate anchor point than one might initially expect.

“Extrinsic mortality,” whose effect on ageing is widely debated (e.g., Wensink et al. 2017), can be thought to influence the result in multiple ways. Equation 2 predicts adult mortality to be negatively correlated and juvenile mortality to be positively correlated with maximum life span across species (increased juvenile mortality corresponds to a decrease in σ). Mortality can also influence the outcome by decreasing $N_{e}$. All else being equal, fecundity is predicted to be negatively correlated with maximum life span (eqs. (2), (3)), indicating that care must be taken when using between‐species comparisons to infer that organisms face a trade‐off between life span and reproduction. Although a negative fecundity‐life span correlation could arise from such a physiological trade‐off, equation 2 shows that a negative correlation at the interspecies level is expected even in the absence of one, instead arising from the simple assumption of population stationarity (see also Cohen et al. 2020 for a similar point and critical discussion on trade‐offs in intra‐ and interspecific comparisons). It is worth reiterating that equations (2), (3) do not assume any particular values for adult mortality, juvenile survival, or fecundity. Instead, they only assume that these values must have been in balance over very long evolutionary timescales in the past, where the population must have been close to stationary on average (Fisher 1930; Hamilton 1966; Charlesworth & Williamson 1975). It is the evolutionary outcome of these long timescales that we retrospectively examine when we observe maximum life spans in nature. The equations relate this outcome to the drift barrier.

Equation 2 is also tentatively in line with the observation that estimated age at onset of senescence is strongly positively correlated with generation time in birds and mammals (Jones et al. 2008). Generation time for the life history used in derivation of equation 2 is $T=b+\frac{1}{\mu }=b+\frac{1}{\sigma f}$ (Lehtonen & Lanfear 2014), which differs from equation 2 only by the factor $\ln(N_{e})$ in the second term. Although equation 2 relates explicitly to maximum life span rather than age at onset of senescence, the two are likely to often be positively correlated (maximum life span can obviously not be smaller than age of onset, and the two coincide in the simplified hypothetical scenario where only lethal mutations are considered). Overall, equations (2), (3) are transparent in the correlations they predict between maximum life span and model parameters. The equations should be considered as a whole, and care needs to be taken in testing for correlations with individual parameters. For example, the equations predict a positive correlation between maximum life span and effective population size, all else being equal. However, $N_{e}$ is also likely to be correlated positively with fecundity across species: larger animals with smaller populations tend to have lower rates of reproduction. The positive effect of $\ln(N_{e})$ could therefore be compensated for or outweighed by the negative effect of fecundity in the denominator in an across‐species comparison. But importantly, the model goes beyond correlational predictions. It makes explicit quantitative predictions about maximum life span based on simple life history and population parameters, something that previous theory has had limited ability to do. Although equations (2), (3) do not by themselves address the evolution of gradual senescence, they capture the “pace of ageing” and the variation in the time‐scale on which mortality progresses across species (Baudisch 2011).

Whether examining correlational or quantitative aspects of the theory, it is important be aware of what equations (2), (3) are saying. Most importantly, they are not predictions of expected life span. Instead, they are predictions of maximum life span and the limits to natural selection, and how that limit in turn relates to life history and population size. An alternative way to view this is that equations (2), (3) quantify where the “selection shadow” begins in full force (Fig. 2). However, there is an interesting connection between expected life span and maximum life span in the continuous reproduction model: the second term of equation 2 can be written as $\frac{1}{\mu }\ln\left(N_{e}\right)$. Noting that $\frac{1}{\mu }$ equals expected adult life span in a nonsenescent population, one interpretation of equation 2 is that under this life history model, selection can maintain up to $\ln(N_{e})$ mean adult life spans in the face of age‐specific, deleterious mutations (for mammals, typically 7–14 mean adult life spans; see above). Hence, if we estimated average adult life span under natural conditions in a population matching the model assumptions, the result would correspond to approximately $\frac{1}{\mu }$, whereas the longest measured adult life span in a very large sample would approximately correspond to $\frac{1}{\mu }\ln\left(N_{e}\right)$.

### EMPIRICALLY TESTABLE PREDICTIONS

The models considered here suggest possible empirical tests. As a preliminary and illustrative example, it is helpful to consider estimates for the parameters of equation 2: a small rodent‐like example (e.g., house mouse) with b=0.2, f=20, σ=0.2, and $N_{e}=580,000$; a small bat‐like species (e.g., Brandt's bat) with b=1, f=0.5, σ=0.5, and $N_{e}=250,000$; and a large whale‐like example (e.g., blue whale) with b=6, f=0.2, σ=0.5, and $N_{e}=80,000$ (see e.g., Roman and Palumbi 2003 for estimates of historical effective population sizes for large whales, Halligan et al. 2010 for effective population size of wild mice, and Bhak et al. 2017 for *Myotis* bats). Here, the units of *b* and *f* are years and female offspring per year, respectively. Substituting these values into equation 2 yields 3.5 years, 51 years, and 119 years, respectively, which are relatively near observed maximum longevities for similar species (4, 41, and 110 years, respectively, for the house mouse, Brandt's bat, and blue whale; de Magalhães & Costa 2009). It is notable that body size does not enter into the analysis in any way, despite the approximately 20 million‐fold difference in weight between Brandt's bat and the blue whale. This is an intriguing result, given that bats are known to be extremely long‐lived relative to their size, whereas mice of similar size are rather short‐lived, yet all the examples above are results of the same, relatively simple model. It must be emphasized that these results are an illustrative example only, and not intended to be a test of the theory. Nevertheless, they do demonstrate two things: that the theory can produce results in the observed mammalian range from minimal but realistic data, and that it is in principle straightforward to put the theory to empirical test using a much larger dataset. A challenge for rigorous comparative tests of theory will be sourcing data that corresponds to the distant evolutionary past and is not influenced by human intervention. For example, contemporary survival and population size data collected for conservation purposes may be misleading in this context. However, *b* and *f* can be considered intrinsic traits of the organism, more robustly reflecting values that have determined selection in the distant past.

An empirical question that has already been partially answered is whether life span scales with effective population size, as suggested by equations (2), (3). This question was addressed in a study by Lohr et al. (2014), who compared rates of ageing and longevity in populations of *Daphnia magna*. Lohr et al. also developed a model to account for finite population effects, but the model was framed in terms of mutations of small effect, very different from the model presented here with mutations of large effect related to the drift barrier. Nevertheless, the empirical results of Lohr et al. confirmed the expectation that life span scales with population size. Because the current model is framed in terms of effective population size (rather than census population size), a further prediction is that species with more fractured population structures should have shorter life spans than those with continuous population structures (e.g., island versus mainland species), and any process that alters effective population size should in principle have the same effect. For example, species with strong sexual selection may have decreased effective population size, and hence potentially decreased life span relative to similar species where reproduction is more evenly distributed among individuals. Regarding the often‐contested relationship between extrinsic mortality and longevity, equation 2 indicates that juvenile and adult mortality are expected to have opposite effects on the evolution of longevity (in line with the results of Caswell & Shyu 2017) and should therefore be analysed separately.

### NEGLIGIBLE SENESCENCE

**Figure 3:**
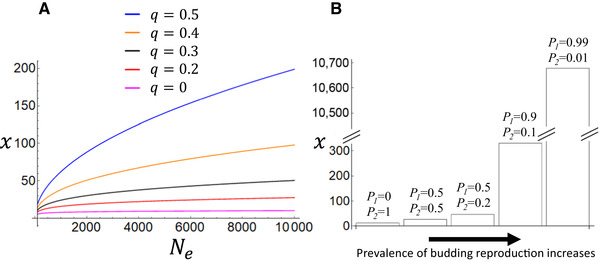
Evolution of extraordinary life spans. In the analytical results of panel A, extrinsic mortality decreases over the adult lifetime, for example, due to indeterminate growth. Age‐specific extrinsic mortality is modeled in this example as $\frac{\mu }{1+q(\tau -b)}$, where τ is age and *q* is a parameter that determines the strength of age‐specific extrinsic mortality decrease. Repeating the derivation of equation 2 with this assumption obtains $x=b+\frac{{N_{e}}^{\frac{q}{\sigma f}}-1}{q}$ (see Supporting Information for derivation). The stronger the decrease in extrinsic mortality with age, the higher the temporal drift barrier and evolving maximum life span *x*. The lowermost line (*q* = 0) corresponds to equation 2 with constant adult extrinsic mortality. In panel B, reproduction takes place partially via outgrowths or fragments of the parent (e.g., budding), and these fragments are assumed to inherit their parents biological age, that is, they are “born older” than offspring originating from gametic reproduction. Budding reproduction alternates with gametic reproduction where age is not inherited. The bars represent different autocorrelations for reproductive mode. *p*_1_ is transition probability from gametic to budding reproduction, whereas *p*_2_ is the transition probability from budding to gametic reproduction. Hence, budding reproduction is absent in the leftmost bar, whereas budding dominates the lifecycle in the rightmost bar. Note the break in the *y*‐axis. The lack of “resetting” of biological age in budding reproduction makes late‐acting mutations visible to selection, and there is no limit to how far life span can evolve as the prevalence of budding increases. Apart from transitions between the two reproductive modes, the simulation used to generate panel B is similar to Figure 1. See Supporting Information for simulation code. In both panels $N_{e}=1600$ and b=σ=f=1.

Recent research has uncovered much diversity in patterns of ageing and suggests that some organisms have negligible senescence (Jones et al. 2014; Jones & Vaupel 2017). Can the present models clarify our understanding of such extreme longevities? The most immediate answer suggested by equations (2), (3) is that species with long juvenile periods, large population sizes, low adult mortality, and low rates of recruitment (arising from low fecundity and/or high juvenile mortality) can all be expected to have long maximum life spans. But more generally, equation 2 and its discrete counterpart can be thought to set a null model for understanding limits to longevity for simple life histories, whereas analyzing special cases can shed light on the diversity seen in nature. Equations (2), (3) arise from a specific life ‐history model that approximates, for example, many mammal species, but the general framework presented here is in agreement with the claim that senescence is not inevitable (Jones & Vaupel 2017). An analogous derivation to that for equation 2 can be applied to alternative life histories. Consider, for example, a species with adult extrinsic mortality risk that decreases with age (e.g., due to indeterminate growth and associated decreased predation risk). Figure 3A illustrates results for this kind of life history for which an analytical equivalent of equation 2 can be derived (see legend to Fig. 3, and derivation in the Supporting Information). The results show that longevity in such an organism can in principle be maintained significantly beyond the ages suggested by equations (2), (3) (the lowest curve in Fig 3A corresponds to eq. 2). Similar examples could be constructed for many alternative life histories, for example, where fecundity increases with age.

Some organisms with clonal reproduction have much reduced senescence, one of the most famous examples being the apparently immortal hydra that reproduce clonally via forming buds as well as via gametes (Martinez 1998; Schaible et al. 2015). Theory suggests that the extreme longevity of clonal organisms could be because clonally produced offspring can begin their life in quite a different developmental stage than those starting their life as gametes (Caswell 1985, Galipaud & Kokko 2020)—in a sense, newborn clones can be “older” than those born via a gametic reproductive pathway. Let us consider an extreme example of this in the context of our model and return to the case of test tubes that carry a stopwatch, started at birth, and connected to a self‐destruct device that shatters the tube when its stopwatch reaches a given reading. What happens if, instead of starting the stopwatch at birth, the parent passes on a stopwatch with an identical reading to its own? The outcome is of course that the readings would accumulate in lineages, eventually any reading would be reached, and all tubes would shatter. In biological terms, any lethal mutation, no matter how late‐acting would be exposed to selection and could not be fixed in the population, resulting in selection maintaining immortality. Clonal reproduction could be a biological counterpart where the stopwatch might not be reset, or is reset only partially. It seems feasible that this could be the case when an offspring is produced from a physical outgrowth of the parent in an essentially unbroken lineage of somatic cells (e.g., budding). In such a case, it seems far from obvious that age would be entirely “reset,” whereas in sexual reproduction development starts anew from gametes after fertilisation. If biological age is carried down a clonal lineage, late‐acting deleterious mutations are exposed to natural selection and extremely high longevities can be maintained. This is simulated in Figure 3B, where gametic reproduction alternates with budding reproduction. The more budding dominates the life cycle, the longer the resulting maximum life span.

Admittedly, this example relies on strong assumptions about differences in clonal reproduction and gametic reproduction, where the latter “rejuvenates” and the former does not. Whether such a dichotomy is true in specific cases remains an open question and is subject to many complicating factors (see Galipaud & Kokko 2020 for a recent discussion), but in extreme cases it seems plausible. We typically take it for granted that a sexually reproduced offspring is born at “age 0” and does not inherit its parents’ age. At the other extreme, making a truly physically exact copy of a parent in every detail would inevitably (by definition of a physically exact copy) pass on any physical factors associated with age. Reproduction by budding may fall somewhere in between these examples, where the offspring begins to develop from a physical fragment of the parent, but is not a physically exact copy in its entirety. The example of Figure 3B is intended to be a proof of principle, rather than an exact model of a specific biological system. It shows that *if* gametic reproduction “rejuvenates” the progeny and budding reproduction does not, then organisms with significant periods of budding reproduction in their lifecycle can maintain arbitrarily long maximum life spans. Conversely and intriguingly, this example suggests that we may be mortal because our “clock is reset” at birth. If such rejuvenation did not take place in newborns, deleterious late‐acting genes would be strongly selected against, preserving a nonsenescent life history.

When the population is divided into castes or classes with longevity evolving independently in different classes, and we are interested in longevity of the reproductive class, adult extrinsic mortality μ in equation 2 must also be of the reproductive class. This can have major implications for maximum life span *x*. For example, in eusocial insects extrinsic mortality of queens can be extremely low, which can result in the evolution of very long queen life spans (Keller & Genoud 1997). In some eusocial organisms, castes are not irreversibly determined, and all females have a chance of becoming the reproductive individual. This is the case, for example, in the eusocial and very long‐lived naked mole‐rat (Ruby et al. 2018). In the context of the present model, the longevity of such species can be explained by the fact that at any given time only the queen in a single colony of many females reproduces, which in turn implies that per‐female birth rate (corresponding to *f* in the model), and hence population turnover rate is low. Therefore maximum life span predicted by equation 2 is again high, consistent with observation. It should, however, be noted that eusocial organisms are likely to have reduced $N_{e}$ (Romiguier et al. 2014), which has a reducing effect on maximum life span. An analysis including estimates of $N_{e}$ will therefore be necessary to confirm whether equation 2 can predict life spans of eusocial organisms.

In summary, an explicit consideration of Medawar's verbal model (Medawar 1952) with mutations of large effect and finite population size exposes a clear and simple connection between the evolution of maximum life span and the drift barrier. This link in turn reveals a quantitative aspect of the evolution of senescence that remains inaccessible with models that assume mutations of small effect and infinite population size. The model predicts maximum life spans that are in the observed range for mammals, and makes clear, empirically testable predictions for comparative studies and potentially also for experimental evolution.

## AUTHOR CONTRIBUTION

JL was the sole contributor to all aspects of this work.

## CONFLICT OF INTEREST

The author declares no conflict of interest.

Associate Editor: K. Lythgoe

## Supporting information

Supplementary Material
