# Supplementary material for: Longevity and the drift barrier: Bridging the gap between Medawar and Hamilton
Source: Evol Lett. 2020 May 24;4(4):382–93. doi: 10.1002/evl3.173 (PMC7403686; doi:10.1002/evl3.173)
Supplement: Supplementary file 1 — Supplementary Material [file EVL3-4-382-s001.pdf]

Supplementary Information for

Longevity and the drift barrier:

Bridging the gap between Medawar and Hamilton

Jussi Lehtonen

The University of Sydney, Faculty of Science, School of Life and Environmental Sciences, Sydney, 2006 New South Wales, Australia.

E-Mail: jussi.lehtonen@iki.fi (corresponding author)

This PDF file includes the following supplementary methods

1) Derivation of equation 3
2) Analytical models for alternative life histories
3) Simulations

Supplementary methods 1: Derivation of equation 3

Equation 3 is the discrete counterpart to equation 2. As in the derivation of equation 2, the life cycle of the organism is divided into two phases: a single maturation phase (phase 1) of length b which a newborn survives with probability σ, after which the newly matured individual enters a pool of adults competing repeatedly for discrete (e.g. seasonal) reproductive opportunities (phase 2). She thereafter reproduces with litter size f per year (or more generally, per time unit defined by the interlitter-interval), and phase 2 continues until she is eventually unsuccessful and dies.

In the ancestral non-senescent population adult survival probablity (p) over each reproductive season is constant, so that probability of survival from new-born to completion of k reproductive seasons is σp^k. A stationary population implies $\sum_{k=1}^{\infty} \sigma p^k f = \frac{\sigma f p}{1-p} = 1$, from which we can solve $p = \frac{1}{1+\sigma f}$. Now consider the fate of a mutant who dies or stops reproducing after y reproductive events if she has survived that far. The expected difference in her lifetime reproductive success relative to the wild-type is

$$s = -\sum_{k=y+1}^{\infty} \sigma p^k f = -\sum_{k=y+1}^{\infty} \sigma (\frac{1}{1+\sigma f})^k f = -(\frac{1}{1+\sigma f})^y \quad \text{(S1)}$$

As with the continuous equation, we solve the limiting case for $|s| < 1/N_e$:

$(\frac{1}{1+\sigma f})^y = 1/N_e$, so that $y = \frac{\ln(N_e)}{\ln(1+\sigma f)}$, and given that y does not include the pre-reproductive period, the corresponding total lifespan is $x = y + b$, or

$$x = b + \frac{\ln(N_e)}{\ln(1+\sigma f)} \quad \text{(S2)}$$

Supplementary methods 2: Analytical models for alternative life histories

The logic of equation 2 is not limited to the specific life history used in its derivation: the functions describing life history can be replaced with alternative functions. Here I consider a life history of particular interest, illuminating the possibility of extended lifespan in indeterminate growers.

Consider a species whose extrinsic mortality decreases with age from maturity onwards, for example due to indeterminate growth and associated diminishing vulnerability to mortality sources. For this illustrative example we model age-specific extrinsic mortality as follows: $u(\tau) = \frac{\mu}{1+q(\tau-b)}$, where τ is age, q is a constant that determines the extent of age-specific decrease, and μ adjusts the overall level of mortality. With q=0 we have constant adult mortality μ as in the main text.

The derivation is analogous to that of equation 2 in the main text.

First, survival probability to age $t>b$:

$$l(t) = \sigma e^{-\int_b^t u(\tau)d\tau} = \sigma e^{-\left(\frac{\mu \log(1-bq+qt)}{q}\right)} = \sigma(1 - bq + qt)^{-\frac{\mu}{q}} \quad \text{(S3)}$$

The condition for a stationary population:

$\int_0^\infty l(t)f(t)dt = \int_b^\infty \sigma(1 - bq + qt)^{-\frac{\mu}{q}} f dt = 1$ which simplifies to $\frac{\sigma f}{\mu - q} = 1$, so we have

$$\mu = \sigma f + q \quad \text{(S4)}$$

Consequently survival in a stationary population is $l(t) = \sigma(1 - bq + qt)^{-\frac{\sigma f+q}{q}}$

and the selection coefficient

$$s = \begin{cases} -\int_b^\infty \sigma(1 - bq + qt)^{-\frac{\sigma f+q}{q}} f dt = -1 & x \leq b \\ -\int_x^\infty \sigma(1 - bq + qt)^{-\frac{\sigma f+q}{q}} f dt = -(1 - bq + qx)^{-\frac{\sigma f}{q}} & x > b \end{cases} \quad \text{(S5)}$$

Now we can solve $|s| = 1/N_e$, or $(1 - bq + qx)^{-\frac{\sigma f}{q}} = 1/N_e$:

$$x = b + \frac{N_e^{\frac{q}{\sigma f}} - 1}{q} \quad \text{(S6)}$$

Figure 2A in the main text is based on equation S6. Taking the limit of S6 as $q \rightarrow 0$ recovers equation 2 in the main text, corresponding to the lowest curve in Figure 2A.

Supplementary methods 3: Simulations

The simulations allow an arbitrary mutation distribution (see Figure S1 for the distributions used) and frequent mutations, with several mutations in the population simultaneously. The simulation also circumvents some assumptions used in the original derivation of mutation fixation probabilities and the drift barrier (Kimura 1962; Li 1978; Kimura 1984), which are at the core of the analytical model. Instead, the results arise organically out of the random simulation process without reference to fixation or the drift barrier. Matlab code for the simulations is provided below. Code for Figure 1 is commented throughout. Additional codes include comments at key points.

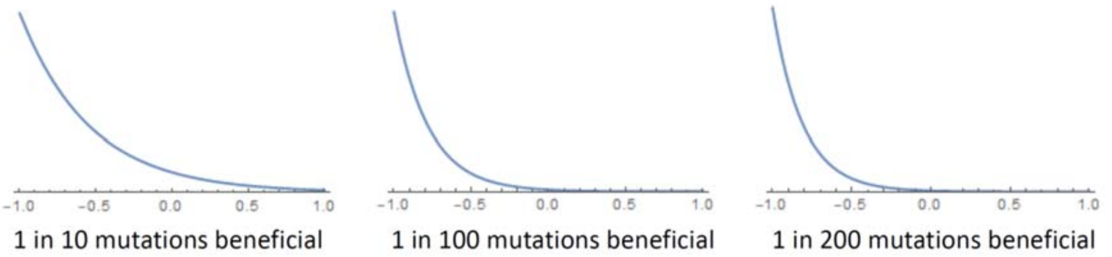

Figure S1. Distribution of mutation effects used in the simulations, reflecting the assumption that only a minority of mutations have a beneficial effect. The gamma distributions (Eyre-Walker & Keightley 2007) have been shifted so the minimum value is -1, and truncated so that values >1 are excluded (different shifts are used in different simulations, as described in the main text). Figure 1 was generated using the middle distribution, while Figure 2 uses all three. The shape parameter for all three panels is 1, and the scale parameter is 0.458, 0.218 and 0.189 respectively from left to right.

Matlab code for Figure 1

```
function [ finalresult ] = LongevityDiscrete( popsize,fec,juvsurv,b,scale)
% Note that this simulation will display the number of elapsed reproductive
% bouts. The time spent as a pre-reproductive (age at first reproduction b) must be added
manually to the
% result.

%maxrun=1e6;                  %How many generations. 1e6 used in 1A
maxrun=4e6;                  %How many generations. 4e6 used in 1B due to large N

howmanyavg=100;              %This and the following line define the number of data points and
the interval between them that are used to display an intermediate result as the simulation
runs
avginterval=1000;
intermediateaverages=zeros(1,howmanyavg); %Vector for computing the intermediate result
mincounter=0.7*maxrun;         %The final result is computed as the average genetically
determined maximum lifespan of the last 30% generations
gentime=b+1/(fec*juvsurv);      %Generation time of the non-senescent phenotype
maxrun=ceil(gentime*maxrun);    %Runtime is adjusted by generation time
mincounter=ceil(mincounter*gentime);  %The final result is the mean of the results from
mincounter onwards

ne=popsize*(1+b*juvsurv*fec)/(1+juvsurv*fec);   %Effective population size as per Hill 1979
predicted=log(ne)/log(1+juvsurv*fec);           %Analytical prediction
startage=round(predicted*1.5);                  %The simulation is started at a maximum
lifespan of 1.5*(analytical prediction). This has no bearing on the result, and the starting
point could be arbitrarily high, but a larger number of generations may be required.

parents=zeros(popsize,2); parents(:,2)=startage; %Initialise parental matrix. Each individual
carries a value for its age, and for the genetically determined maximum lifespan.
juveniles=zeros(popsize*fec*juvsurv,2,b); juveniles(:,2,:)=startage;  %Initialise juvenile
array where juveniles are held until they mature
```

```
finalresult=0;
intermediateaverage=0;
mutprob=1;                    %A mutation appears with certainty in every generation

counter=1;
while counter<maxrun




    if rand<mutprob
        mutatethis=ceil(popsize*rand);
        parents(mutatethis,2)=max(0,parents(mutatethis,2)*(1+min(1,gammut(1,scale))));  %A
randomly picked parent is mutated with mutation picked from gamma distribution with scale
parameter ‘scale’
    end

    offspring=repmat(parents,fec,1);  %Parents reproduce
    offspring(:,1)=0; %Offspring do not inherit their parent's age, which is reset to 0

    if juvsurv<1
        survoffspring=datasample(offspring,round(popsize*fec*juvsurv),'Replace',false);
%Offspring survival
    else
        survoffspring=offspring;
    end


    if b>=2
        juveniles=cat(3,juveniles(:,:,2:end),survoffspring);        %The juvenile maturation
array advances by one year
    else
        juveniles=survoffspring;
    end


    parents(:,1)=parents(:,1)+1;                                   %Parents age by one year
    wgts=round([(parents(:,1)<parents(:,2));(juveniles(:,1,1)<juveniles(:,2,1))]);  %Survival
weightings
    parents=datasample([parents;juveniles(:,:,1)],popsize,'Replace',false,'Weights',wgts);
%Randomly sample the parents of the next generation



    counter=counter+1;
    if counter>mincounter
        finalresult=(finalresult*(counter-mincounter-1)+mean(parents(:,2)))/(counter-
mincounter);       %The final result is the mean of the last 30% generations
    end
    if counter/avginterval==floor(counter/avginterval)
        intermediateaverages=cat(2,intermediateaverages(2:end),mean(parents(:,2)));   %This
tracks an intermediate result which is displayed throughout
        intermediateaverage=mean(intermediateaverages);
    end


    if rand<0.001           %Occasionally shows intermediate results
        [mean(parents(:,2)) finalresult intermediateaverage predicted ]
        [counter maxrun]
    end


end
```

Code for mutations in all simulations (gammut.m)

```
function effect=gammut(shape, scale);
effect=gamrnd(shape,scale)-1;
while effect>1
      effect=gamrnd(shape,scale)-1;
end
```

Examples of population fluctuations in Fig. 1B

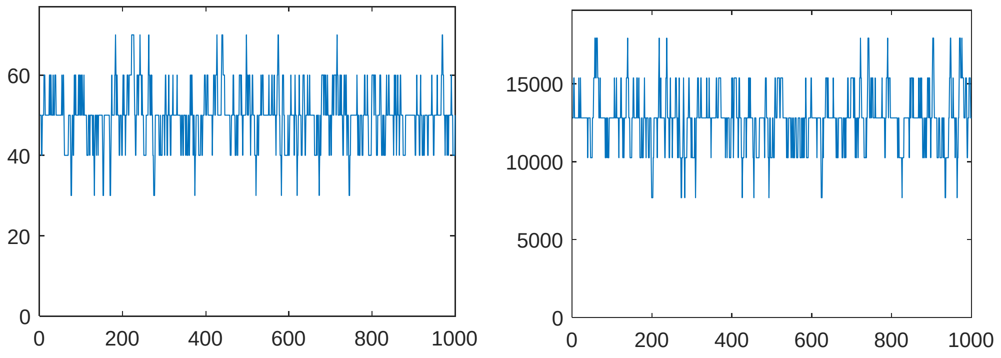

Figure S2. Examples of population size fluctuations in Fig. 1B. The two lowest curves of Fig. 1B include simulation results with fluctuating population size. The two panels show examples of these random fluctuations over 1000 generations, where the adult population sizes fluctuate randomly with long-term mean 50 and 12800 respectively.

Matlab code for population fluctuations in Fig. 1B

```
function [popsize] = pop(currentpop,meanpop,nochange,increment,changemultiplier)

p_nochange=nochange*(1-abs(currentpop/meanpop-1));

if rand<p_nochange
    popsize=currentpop;
else
    if currentpop>meanpop
        p_decrease=0.5+changemultiplier*0.5*(currentpop/meanpop-1);
        p_increase=1-p_decrease;
    else
        p_increase=0.5+changemultiplier*0.5*(1-currentpop/meanpop);
        p_decrease=1-p_increase;
    end
    if rand<p_increase
        popsize=currentpop+increment;
    else
        popsize=currentpop-increment;
    end
end


end
```

Matlab code for Figure 2

```
function longtermmeancum = senesce( popsize,fec,juvsurv,scale)

longtermmeancum=0;

mp=50;       %This is the number of years that are exposed to
%mutations in the simulation. Some limit has to be set, or else we would be
%selecting the year of onset from an infinite range. Individuals are
%nevertheless allowed to live arbitrary long periods. Furthermore, given
%that in all the simulated examples senescence evolves to be such
%that death occurs well before age 50, this upper limit does not compromise
%the results.

maxrun=1e6;
gentime=1+1/(fec*juvsurv);
time=ceil(gentime*maxrun);
prediction=log(popsize)/log(1+fec*juvsurv);
tester=round(time/100);
counter=1;
parents=ones(popsize,mp+1);
longevity_temp=mp;
longtermmean=zeros(1,mp);
longtermcumu=zeros(1,mp);
meansmatrix=zeros(1000,mp);
meanlongevity=0;
mincounter=round(0.7*time);
meanlongevitycounter=0;
for i=1:time

    if rand<1    %A mutation in every generation
        mutatethis=ceil(popsize*rand);       %Who mutates
        mutateyear=ceil(mp*rand);            %Which is the first year affected
        effect=gammut(1, scale);             %Magnitude of the mutation effect
        window=round(0.5*mp*rand);           %Length of time window during which mutation has
an effect
            minmut=max(2,mutateyear);
            maxmut=min(mp+1,mutateyear+window);

parents(mutatethis,(minmut):(maxmut))=min(1,max(0,(effect+parents(mutatethis,(minmut):(maxmut)
))));



    end

    offspring=[];
    for j=1:fec
        offspring=[offspring;parents];
    end
    offspring(:,1)=1;

    if juvsurv<1
        survoffspring=datasample(offspring,round(popsize*fec*juvsurv),'Replace',false);
    else
        survoffspring=offspring;
    end
    offspring=[survoffspring;[parents(:,1)+1,parents(:,2:end)]];

    for j=1:(round(fec*juvsurv*popsize)+popsize);
        if offspring(j,1)<mp+1
            wgts(j)=offspring(j,offspring(j,1)+1);
        else
            wgts(j)=1;
        end
    end

    parents=datasample(offspring,popsize,'Replace',false,'Weights',wgts);
    %The main difference here to Fig. 1 is that now individuals can
    %have arbitrary age-specific 'competitive weights' between 0 and 1,
    %and it is these weights that are altered by mutation. In Fig. 1,
    %the weight is always either 0 or 1, and those with a 0 weight
    %always die. In other words, in Fig. 1, mutations are lethal at a
    %fixed age, whereas in Fig. 2 individuals can senesce more
    %gradually, with decreasing competitive ability and increasing
    %chance of death at population regulation as they age.
```

```
    if i>mincounter
        cumu=cumprod(parents(:,2:end));
        meancum=mean(cumu);
        longtermmeancum=longtermmeancum*(i-mincounter-1)/(i-mincounter)+meancum/(i-
mincounter);
    end

    if i/tester==floor(i/tester)

        meansenesce=mean(parents(:,2:end),1);
        meansmatrix=cat(1,meansmatrix(2:end,:),meansenesce);

        longtermmean=((counter-1)*longtermmean+meansenesce)/counter;

        cumu=cumprod(meansenesce(1:end));
        longtermcumu=((counter-1)*longtermcumu+cumu)/counter;
        1:mp;
        longevity_temp = find(cumu<0.0001,1);
        if isempty(longevity_temp)
            longevity(counter)=mp;
        else longevity(counter)=longevity_temp;
        end

        if i>mincounter
            meanlongevitycounter=meanlongevitycounter+1;
            meanlongevity=meanlongevity*(meanlongevitycounter-
1)/(meanlongevitycounter)+longevity(counter)/(meanlongevitycounter)
        end

        meanage(counter)=sum(cumu);
        [i/time meanage(counter) longevity(counter) prediction]
        counter=counter+1;
    end

end
temp=mean(parents); age=temp(2);
cumprod(parents(1,2:end),2)
parents(1,2:end)
figure; plot(longtermcumu);
figure; plot(mean(meansmatrix));
age=meanlongevity;
[prediction age]
End
```

Matlab code for Figure 3B

```
function [ finalresult ] = LongevityDiscrete_BuddingAutocorrelation(
popsize,fec,juvsurv,b,gametictobudding,buddingtogametic)
% Note that this simulation will display the number of elapsed reproductive
% bouts. The time spent as a pre-reproductive (age at first reproduction b) must be added
manually to the
% result.

%gametictobudding,buddingtogametic are transition probabilities from
%gametic reproduction to budding and vice versa

maxrun=1e6;
howmanyavg=100;
avginterval=1000;
intermediateaverages=zeros(1,howmanyavg);
mincounter=0.7*maxrun;
gentime=b+1/(fec*juvsurv);
maxrun=ceil(gentime*maxrun);
mincounter=ceil(mincounter*gentime);

ne=popsize*(1+b*juvsurv*fec)/(1+juvsurv*fec);
predicted=log(ne)/log(1+juvsurv*fec);
startage=round(predicted*1.5);

parents=zeros(popsize,2); parents(:,2)=startage;
```

```
juveniles=zeros(popsize*fec*juvsurv,2,b); juveniles(:,2,:)=startage;


finalresult=0;
intermediateaverage=0;

counter=1;


gameticreproduction=1;   %The simulation starts with gametic reproduction


while counter<maxrun


    if rand<1          %Mutation in every generation
        mutatethis=ceil(popsize*rand);
        parents(mutatethis,2)=max(0,parents(mutatethis,2)*(1+min(1,gammut(1, 0.218))));
    end

    offspring=repmat(parents,fec,1);
    if gameticreproduction==1  %Offspring age is reset to 0 in gametic reproduction but not in
budding reproduction
        offspring(:,1)=0;
    end
    if gameticreproduction==0        %Transitions from budding to gametic reproduction
        if rand<buddingtogametic
            gameticreproduction=1;
        end
    end
    if gameticreproduction==1        %Transitions from gametic to budding reproduction
        if rand<gametictobudding
            gameticreproduction=0;
        end
    end


    if juvsurv<1
        survoffspring=datasample(offspring,round(popsize*fec*juvsurv),'Replace',false);
    else
        survoffspring=offspring;
    end


    if b>=2
        juveniles=cat(3,juveniles(:,:,2:end),survoffspring);
    else
        juveniles=survoffspring;
    end


    parents(:,1)=parents(:,1)+1;
    wgts=round([(parents(:,1)<parents(:,2));(juveniles(:,1,1)<juveniles(:,2,1))]);
    parents=datasample([parents;juveniles(:,:,1)],popsize,'Replace',false,'Weights',wgts);



    counter=counter+1;
    if counter>mincounter
        finalresult=(finalresult*(counter-mincounter-1)+mean(parents(:,2)))/(counter-
mincounter);
    end
    if counter/avginterval==floor(counter/avginterval)
        intermediateaverages=cat(2,intermediateaverages(2:end),mean(parents(:,2)));
        intermediateaverage=mean(intermediateaverages);
    end


    if rand<0.001
        [mean(parents(:,2)) finalresult intermediateaverage predicted ]
        [counter maxrun]
    end

    end
```

References for supplementary information

Eyre-Walker, A. & Keightley, P.D. (2007). The distribution of fitness effects of new mutations. *Nat. Rev. Genet.*, 8, 610-618.

Kimura, M. (1962). On the probability of fixation of mutant genes in a population. *Genetics*, 47, 713.

Kimura, M. (1984). *The Neutral Theory of Molecular Evolution*. Cambridge University Press.

Li, W.-H. (1978). Maintenance of genetic variability under the joint effect of mutation, selection and random drift. *Genetics*, 90, 349-382.
